# A systematic nomenclature for mammalian tropomyosin isoforms

**DOI:** 10.1007/s10974-014-9389-6

**Published:** 2014-11-05

**Authors:** Michael A. Geeves, Sarah E. Hitchcock-DeGregori, Peter W. Gunning

**Affiliations:** 1 School of Biosciences, University of Kent, Canterbury, CT2 7NJ UK; 2 Robert Wood Johnson Medical School, Rutgers University, Piscataway, NJ 08854 USA; 3 School of Medical Sciences, University of New South Wales (UNSW), Sydney, NSW 2052 Australia

**Keywords:** Cytoskeleton, Actin binding protein, Muscle thin filament

## Abstract

Tropomyosin, a ubiquitous protein in animals and fungi, is associated with the actin cytoskeleton and is involved with stabilising actin filaments and regulating the interaction of the filament with other actin binding proteins. The protein is best known for its role in regulating the interaction between actin and myosin in muscle contraction but in recent years its role as a major player in the organisation and dynamics of the cytoskeleton has been increasingly recognised. In mammals Tpm is expressed from four distinct genes and alternate splicing of each gene can produce a total of up to 40 different mRNA variants most of which are expressed as proteins. We are expecting a renaissance in the study of tropomyosins as the roles of these different isoforms are beginning to be deciphered. However, it is our belief that such a renaissance is being limited by confusion over the naming systems for the tropomyosin isoforms. These result in even experienced workers struggling to reconcile work done in different laboratories and at different times. We propose here a systematic nomenclature for tropomyosin based on the best current practice. We recommend the adoption of these names and a cross-reference to the table of alternate names and accession numbers for protein sequences is included here. The National Center for Biotechnology Information (NCBI) website has been amended to include the nomenclature for the human, mouse and rat genes.

Tropomyosin (Tpm) is a protein family associated with the stabilisation and regulation of the actin cytoskeleton (Gunning et al. 2008). It is expressed in opisthokonts including animals and fungi, but is not documented in plants, protists or procaryotes (Barua et al. 2011; Cranz-Mileva et al. 2013). Tpm polymerises head to tail as a two-chained coiled coil along both sides of a helical actin filament stabilising the actin structure and interacting with a range of other actin binding proteins to regulate the actin cytoskeleton. The protein was discovered in the 1940s as a component of the actin filaments of striated muscle (Bailey 1946) and this remains the best studied example of Tpm function. In striated muscle, together with troponin, it enables calcium regulation of striated muscle contraction by blocking (−Ca^2+^) or allowing (+Ca^2+^) myosin access to its binding site on actin (Gordon et al. 2000; Geeves 2012). Since the 1940s the different possible roles of Tpm and the number of interacting partners has multiplied (see special issue of J Muscle Res. Cell Motil., Marston & Gautel 2013). We now know that in mammalian cells Tpm is expressed from four separate genes and alternate promoter selection and splicing of each gene can produce up to 40 different mRNA variants most of which have been shown to be expressed as proteins in different tissues (Pittenger et al. 1994; Vindin and Gunning 2013).

The steady discovery of different splice variants in specific tissues has, over the years, led to a proliferation of different names for the isoforms, some identified with specific tissues but later found with a broader distribution. This has resulted in a great deal of confusion in the literature and it is often difficult, even for those of us in the field, to be sure which isoforms we are each referring to. It must be an even bigger nightmare for those trying to enter the field. The lack of a coherent nomenclature is an impediment to progress. We firmly believe that Tpm research is due for a renaissance from both the structure–function perspective in muscle research and the cytoplasmic perspective in the localisation-function of distinct isoforms and their binding partners. This is evidenced by the collection of articles published in a special issue of J Muscle Res Cell Motil devoted to Tpm Marston & Gautel (2013). This renaissance will be enhanced if we can establish a widely agreed-upon nomenclature that the majority of active researchers will use. It will be much better if we can establish this now as the field begins to expand and other pressures develop on the nomenclature.

After consulting colleagues working with Tpm we now make a specific proposal for how the nomenclature could be simplified and rationalised and to make it compatible with current best practice for gene and protein names. The proposal keeps as close as possible to some well-established historical precedents.

Our suggestion is that any publication dealing with the tropomyosin protein should designate, at the first use, the formal name of the protein isoform as proposed here, and then define the short form or abbreviation that will be used thereafter in the article text. Reference to Tables 1, 2, 3 and 4 here will allow cross-reference to any earlier versions of the names used e.g., α-fast skeletal tropomyosin is Tpm1.1.

**Table 1:** List of tropomyosin isoforms derived from the human TPM1 gene (geneID:7168), the mouse *Tpm*1 gene(geneID:22003) or the rat *Tpm*1 gene (geneID:24851). Gene structure

Short name	Common names	Alternate names in use	Formal protein name	Accession numbers Full length sequences only^a^	Exon usage^b^
Tpm1.1	Tm skα	αTm, fast skTm , cardiac Tm, striated Tm	Tpm1.1st (a.b.b.a)	Human: NP_001018005.1 Mouse: NP_001157720.1 Rat: NP_001288265.1	
Tpm1.2	Tm skα1	κTm, Tmskα1-1	Tpm1.2st (a.a.b.a)	Human: NP_001288173.1 Mouse: none Rat: NP_001288271.1	
Tpm1.3	Tm smα-1	α smooth Tm	Tpm1.3sm (a.a.a.d)	Human: NP_001018020.1 Mouse: none Rat: NP_001029243.1	
Tpm1.4	Tm smα	Tm6, α smooth Tm Tm α1.2	Tpm1.4sm (a.a.b.d)	Human: NP_001018007.1 Mouse: NP_001157721.1 Rat: AAA21804.1	
Tpm1.5	Tm3-1	−	Tpm1.5cy (a.b.a.a)	Human: NP_000357.3 Mouse: NP_001157723.1 Rat: none	
Tpm1.6	Tm2	−	Tpm1.6cy (a.b.b.d)	Human: NP_001018004.1 Mouse: NP_077745.2 Rat: NP_001029241.1	
Tpm1.7	Tm3	Tm 1.4	Tpm1.7cy (a.b.a.d)	Human: NP_001018006.1 Mouse: NP_001157722.1 Rat: NP_001029242.1	
Tpm1.8	Tm5a	−	Tpm1.8cy (b.-.b.d)	Human: NP_001288218.1 Mouse: NP_001157724.1 Rat: NP_001029245.1	
Tpm1.9	Tm5b	−	Tpm1.9cy (b.-.a.d)	Human: none Mouse: NP_001157725.1 Rat: NP_001029246.1	
Tpm1.10	TmBr1	−	Tpm1.10br (a.b.b.c)	Human: none Mouse: NP_001157727.1 Rat: NP_001029244.1	
Tpm1.11	TmBr2	−	Tpm1.11br (b.-.b.b)	Human: none Mouse: none Rat: NP_062004.1	
Tpm1.12	TmBr3	Tmbrα, Tmbrα-1 Tm α-1.6	Tpm1.12br (b.-.b.c)	Human: NP_001018008.1 Mouse: NP_001157728.1 Rat: NP_001288665.1	
Tpm1.13	−	−	Tpm1.13 (b.-b.a)	Human: none Mouse: NP_001157726.1 Rat: NP_001029247.1	

^a ^The accession numbers are from September, 2014 and for full-length human, mouse and rat protein sequences that have been documented at the nucleic acid level. RefSeq numbers are listed when available. The N-terminal Met is removed post translation in all exon 1b-encoded sequences. As far as is currently known the N-terminal amino acid is N-acetylated post translation in all isoforms. NCBI website has been updated to include the above terminology

^b ^The exon notation includes only the protein-coding exons and does not reflect splice variants in non-coding regions at the 3′ end of the mRNA transcripts

**Table 2:** List of tropomyosin isoforms derived from the human TPM3 gene (geneID:7170), the mouse *Tpm*3 gene (geneID:59069) or the rat *Tpm*3 gene (geneID:117557). Gene structure:

Short name	Common names	Alternate names in use	Formal protein name	Accession numbers Full length sequences only^a^	Exon usage^b^
Tpm3.12	Tm skα-slow	$\gamma$Tm αsTm1, slow skTm	Tpm3.12st (a.b.b.a)	Human: NP_689476.2 Mouse: NP_001280677.2 Rat: NP_001288214.1	
Tpm3.13	−	−	Tpm3.13cy (a.b.a.d)	Human: none Mouse: NP_071709.2 Rat: none	
Tpm3.1	Tm5NM1	−	Tpm3.1cy(b.-.a.d)	Human: NP_705935.1 Mouse: NP_001240667.1 Rat: NP_775134.1	
Tpm3.2	Tm5NM2	−	Tpm3.2cy(b.-.b.d)	Human: NP_001036816.1 Mouse: NP_001240669.1 Rat: none	
Tpm3.3	Tm5NM3	−	Tpm3.3cy(b.-.b.a)	Human: EAW53237.1 Mouse: none Rat: NP_001288215.1	
Tpm3.4	Tm5NM4	−	Tpm3.4cy (b.-.b.c)	Human: NP_001036818.1 Mouse: NP_001280678.1 Rat: NP_476556.2	
Tpm3.5	Tm5NM5	−	Tpm3.5cy(b.-.a.a)	Human: NP_001265118.1 Mouse: NP_001258693.1 Rat: none	
Tpm3.7	Tm5NM7	−	Tpm3.7cy (b.-.a.c)	Human: NP_001036817.1 Mouse: none Rat: none	
Tpm3.8	Tm5NM8	−	Tpm3.8cy (b.-.a.a/c)^c^	Human: none Mouse: none Rat: none	
Tpm3.9	Tm5NM9	−	Tpm3.9cy (b.-.b.a/c)^c^	Human: none Mouse: none Rat: none	

^a ^The accession numbers are from September, 2014 and for full-length human, mouse and rat protein sequences that have been documented at the nucleic acid level. RefSeq numbers are listed when available. The N-terminal Met is removed post translation in all exon 1b-encoded sequences. As far as is currently known the N-terminal amino acid is N-acetylated post translation in all isoforms. NCBI website has been updated to include the above terminology

^b ^The exon notation includes only the protein-coding exons and does not reflect splice variants in non-coding regions at the 3′ end of the mRNA transcripts

^c ^The designation a/c indicates that the ninth exon includes exon 9a and five amino acids from exon 9c

**Table 3:** List of tropomyosin isoforms derived from the human TPM2 gene (geneID:7169), the mouse *Tpm*2 gene (geneID:22004) or the rat Tpm2 gene (geneID:500450)^a^. Gene structure

Short name	Common names	Alternate names in use	Formal protein name	Accession numbers Full length sequences only^b^	Exon usage^c^
Tpm2.1	Tm smβ	βTm Tm1, Tm1β, smooth Tm, smooth α-Tm	Tpm2.1sm/cy (a.b.a.d)	Human: NP_998839.1 Mouse: NP_001264805.1 Rat: NP_001019516.1	
Tpm2.2	Tm skβ	β skeletal Tm, β cardiac Tm	Tpm2.2st (a.b.b.a)	Human: NP_003280.2 Mouse: NP_033442.2 Rat: NP_001288164.1	
Tpm2.3		−	Tpm2.3 (a.b.b.d)	Human: NP_001288155.1 Mouse: NP_001264804.1 Rat: none	
Tpm2.4		−	Tpm2.4 (a.b.a.a)	Human: NP_001288156.1 Mouse: none Rat: none	

^a ^The TPM2 genes in mouse, rat and human do not contain exon 1b. It is included here as it is found in chicken and zebrafish

^b ^The accession numbers are from September, 2014 and for full-length human, mouse and rat protein sequences that have been documented at the nucleic acid level. RefSeq numbers are listed when available. The N-terminal Met is removed post translation in all exon 1b-encoded sequences. As far as is currently known the N-terminal amino acid is N-acetylated post translation in all isoforms. NCBI website has been updated to include the above terminology

^c ^The exon notation includes only the protein-coding exons and does not reflect splice variants in non-coding regions at the 3′ end of the mRNA transcripts

**Table 4:** List of tropomyosin isoforms derived from the human TPM4 gene (geneID:7171), the mouse *Tpm*4 gene (geneID:326618) or the rat gene *Tpm*4 (geneID:248512)^a^. Gene structure

Short name	Common names	Alternate names in use	Formal protein name	Accession numbers Full length sequences only^b^	Exon usage^c^
Tpm4.1	Tm4HMW	$\delta$Tm	Tpm4.1cy (a.b.b.d)	Human: NP_001138632.1 Mouse: none Rat: none	
Tpm4.2	Tm4	−	Tpm4.2cy (b.-.b.d)	Human: NP_003281.1 Mouse: NP_001001491.1 Rat: NP_036810.1	

^a ^The TPM4 gene of humans (but not rat or mouse) contains exon 9a but there are no reported Tpm4 isoforms expressing exon 9a. Exon 9a is present in other vertebrates e.g., chicken, frog and zebrafish

^b ^The accession numbers are from September, 2014 and for full-length human, mouse and rat protein sequences that have been documented at the nucleic acid level. RefSeq numbers are listed when available. The N-terminal Met is removed post translation in all exon 1b-encoded sequences. As far as is currently known the N-terminal amino acid is N-acetylated post translation in all isoforms. NCBI website has been updated to include the above terminology

^c ^The exon notation includes only the protein-coding exons and does not reflect splice variants in non-coding regions at the 3′ end of the mRNA transcripts

## Detailed proposals

1. The human tropomyosin genes should be known as *TPM* *1* through *TPM* *4* (*Tpm* *1* through *Tpm* *4* for mouse and rat tropomyosin) to be consistent with other gene nomenclatures.
2. The protein short name Tm is historically well established but Tpm is consistent with standard protein nomenclature and is therefore preferred in the formal name.
3. Tpm1 or Tpm2 specifies the protein is from gene *TPM1* or *TPM2* etc.
4. Alternate isoforms are numbered systematically from one (e.g., Tpm1.1 etc.), keeping as close as possible to any precedent. Thus Tpm2.3 is the tropomyosin isoform from gene 2 isoform 3.
5. In the formal name a subscript designates the tissue most closely associated, *historically*, with the protein isoform. Many such isoforms have been since found in multiple tissues and such designations are not always useful.
  St striated muscle, cardiac/skeletal tissue
  Sm smooth muscle,
  Br brain
  Cy other cytoplasmic
6. The splicing of the four exons that vary in vertebrates can be designated by a four letter code; a.a.b.d indicates exon 1 is splice form a, exon 2 is splice form a, exon 6 is splice form b and exon 9 is splice form d.
  A dash as in b.-.b.d indicates that exon 2 is missing and therefore this is a short form of Tpm.
  Previous publications of Tpm isoforms have indicated that exon 9 can be a combination of two splice forms at the mRNA level (e.g., Vindin and Gunning 2013) but these reflect splice variants in non-coding regions at the 3′ end of the mRNA transcripts. We have therefore omitted such isoforms from our list of expressed proteins.
7. The formal names of the two tropomyosins from the *TPM* *1* gene, found in the contractile filaments of smooth muscle, are then
  Tpm1.3_Sm(a.a.b.d)_ and Tpm1.4_Sm(a.a.a.d)_

The short form of the name would be Tpm1.3 and Tpm1.4

Tables 1, 2, 3 and 4 list the current, known Tpm protein isoforms and the proposed formal names. As far as possible the alternate names for the same protein that have been used in the past are included in columns two and three. We also include, in Table 5, the full set of human Tpm amino acid sequences in the order that the exons appear in the gene. This will allow any ambiguity in the literature to be checked against the names and exon sequences used here. A fuller list of animal Tpm sequences can be found in Barua et al. 2011 and Schevzov et al. 2011.

**Table 5:** Human tropomyosin amino acid sequences in order of the exons in the gene

For additional information about tropomyosin sequences and exon organization in animals, refer to Barua et al. 2011 and Schevzov et al. 2011

It is almost certain that additional isoforms of tropomyosin will be identified in these species. Consideration of all possible splicing combinations already established in at least one isoform leads to a calculation of 48 possible distinct isoforms in humans without consideration of currently unknown splicing alternatives. For example, Cooley and Bergtrom (2001) identified an increased number of potential isoforms from the *TPM1* gene using RT-PCR. Our ability to detect and confirm additional isoforms will depend on both the extent of expression across, and the levels of expression in, different cell types.

The NCBI accession numbers for each isoform are included in Tables 1, 2, 3 and 4 and the human, mouse and rat genes on the NCBI website have been amended to use the nomenclature as listed here.
